# *EPS8*, encoding an actin-binding protein of cochlear hair cell stereocilia, is a new causal gene for autosomal recessive profound deafness

**DOI:** 10.1186/1750-1172-9-55

**Published:** 2014-04-17

**Authors:** Asma Behlouli, Crystel Bonnet, Samia Abdi, Aïcha Bouaita, Andrea Lelli, Jean-Pierre Hardelin, Cataldo Schietroma, Yahia Rous, Malek Louha, Ahmed Cheknane, Hayet Lebdi, Kamel Boudjelida, Mohamed Makrelouf, Akila Zenati, Christine Petit

**Affiliations:** 1 Laboratoire de Biochimie Génétique, Service de Biologie - CHU de Bab El Oued, Université d’Alger 1, Alger, Algérie; 2 INSERM UMRS1120, UPMC, Institut de la Vision, Paris, France; 3 Génétique et Biologie, Centre Hospitalier universitaire de Blida, Université Saad Dahleb, Blida, Algérie; 4 Unité de Génétique et Physiologie de l’Audition, INSERM UMRS1120, Institut Pasteur, Paris, France; 5 Service de Biochime et de Biologie Moléculaire, Hôpital Armand Trousseau, APHP, Paris, France; 6 Service ORL, Centre Hospitalier universitaire de Blida, Blida, Algérie; 7 Service Ophtalmologie, Centre Hospitalier universitaire de Blida, Blida, Algérie; 8 Collège de France, Paris, France

**Keywords:** Epidermal growth factor receptor pathway substrate 8, Congenital deafness, Whole-exome sequencing, Stereocilia bundle, Actin dynamics

## Abstract

**Background:**

Almost 90% of all cases of congenital, non-syndromic, severe to profound inherited deafness display an autosomal recessive mode of transmission (DFNB forms). To date, 47 causal DFNB genes have been identified, but many others remain to be discovered. We report the study of two siblings born to consanguineous Algerian parents and affected by isolated, profound congenital deafness.

**Method:**

Whole-exome sequencing was carried out on these patients after a failure to identify mutations in the DFNB genes frequently involved.

**Results:**

A biallelic nonsense mutation, c.88C > T (p.Gln30*), was identified in *EPS8* that encodes epidermal growth factor receptor pathway substrate 8, a 822 amino-acid protein involved in actin dynamics. This mutation predicts a truncated inactive protein or no protein at all. The mutation was also present, in the heterozygous state, in one clinically unaffected sibling and in both unaffected parents, and was absent from the other two unaffected siblings. It was not found in 120 Algerian normal hearing control individuals or in the Exome Variant Server database. EPS8 is an F-actin capping and bundling protein. Mutant mice lacking EPS8 (*Eps8*^−/−^ mice), which is present in the hair bundle, the sensory antenna of the auditory sensory cells that operate the mechano-electrical transduction, are also profoundly deaf and have abnormally short hair bundle stereocilia.

**Conclusion:**

This new DFNB form is likely to arise from abnormal hair bundles resulting in compromised detection of physiological sound pressures.

## Introduction

**Figure 1:**
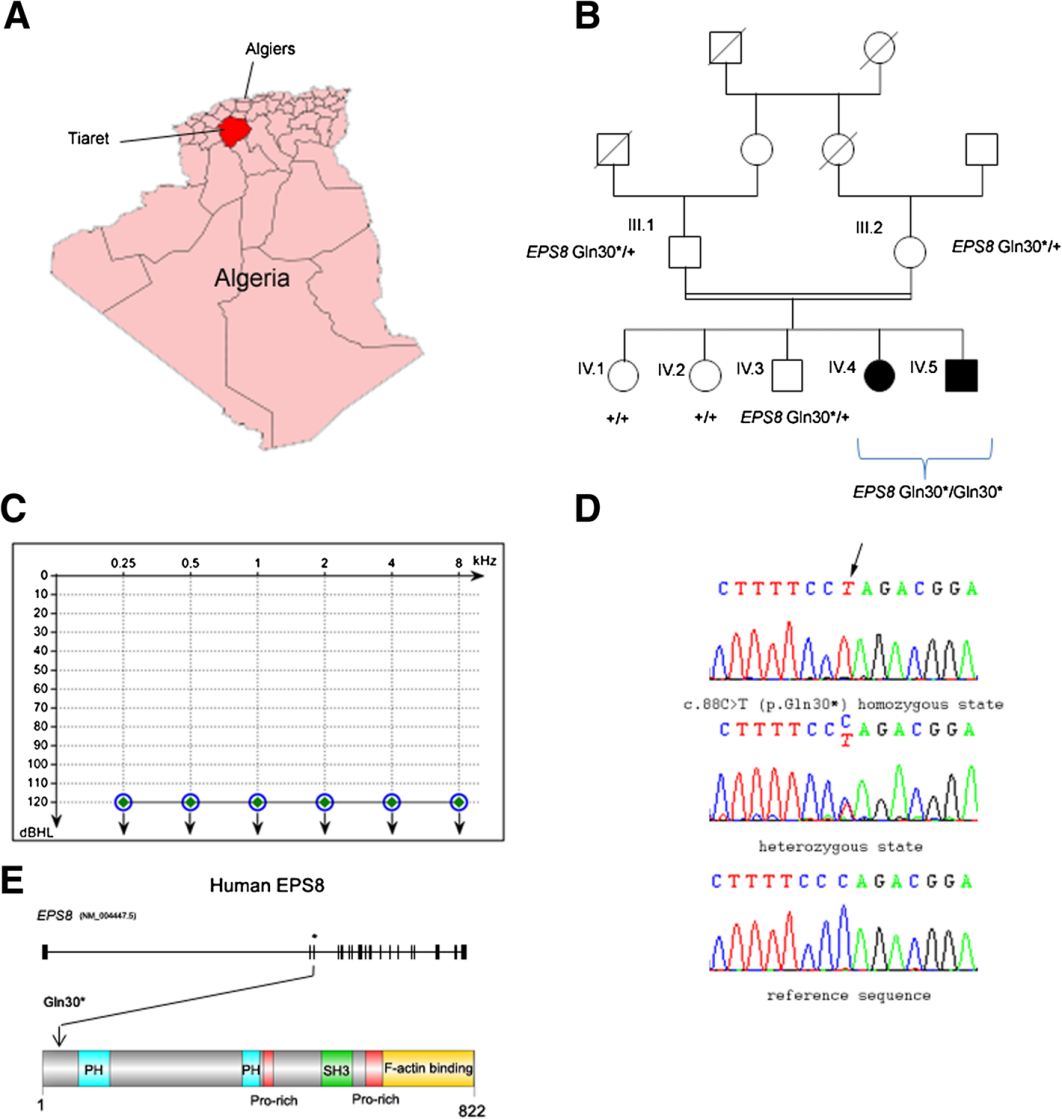
**Clinical and molecular data in the patients harbouring the nonsense mutation in EPS8. (A)** Map of Algeria showing the province of Tiaret (in red). **(B)** Segregation of the nonsense *EPS8* mutation in the family. **(C)** Air-conduction audiometric curves for patients IV.4 (open blue circles) and IV.5 (closed green diamonds) at the ages of 11 and 7 years, respectively. Identical audiometric curves were obtained for both ears in both patients. **(D)** DNA sequencing chromatograms showing the mutation (arrow). **(E)** Schematic representations of the human EPS8 gene and protein. * indicates the position of *EPS8* exon 3. The protein (822 amino acids) contains a split pleckstrin homology (PH) domain (in blue), two proline-rich (Pro-rich) domains (in red), an SRC Homology 3 (SH3) domain (in green), and an F-actin-binding domain (in orange).

In developed countries, congenital hearing impairment is thought to have a genetic origin in more than 70% of cases [1]. In about 90% of all cases of non-syndromic (i.e. isolated), severe to profound congenital inherited deafness, the defect is transmitted in an autosomal recessive manner (autosomal recessive non-syndromic deafness or DFNB forms) [2]. Forty-seven causal genes have been identified for DFNB, but many more remain to be identified. Research of genes involved in severe to profound congenital deafness is particularly efficient in geographic regions in which consanguineous marriages are frequent, such as the southern and eastern Mediterranean Basin, including North Africa [3-5]. We thus collected dozens of families affected by severe to profound deafness from the Algerian province of Tiaret, in the Tell Atlas, to the south-west of Algiers (Figure 1A). We used screening tests for mutations or deletions affecting *GJB2*, which account for 30% to 50% of the congenital profound DFNB cases in countries located around the Mediterranean sea [6], and selected 40 DFNB families without *GJB2* mutations identified, for further analysis. We report here on one of these families, in which we identified a new causative deafness gene.

## Materials and methods

### Patients

This study was approved by the local Ethical Committees and the Committee for the Protection of Individuals in Biochemical Research as required by French legislation. Written consent for genetic testing was obtained from all family members.

### Animals

Mouse cochleas were obtained as previously described [7]. Eyes were collected from adult *Macaca fascicularis* animals housed at the MIRcen platform (CEA/INSERM, Fontenay-aux-Roses, France). Experiments on animals were performed according to protocols approved by the Animal Use Committees of INSERM, CEA (for *Macaca fascicularis*), Institut Pasteur, and the ARVO Statement for the Use of Animals in Ophthalmological and Vision Research.

### Auditory tests

All family members underwent pure-tone audiometry in a sound-proof room, with recording of air-conduction and bone-conduction thresholds. Air-conduction pure-tone average (ACPTA) threshold in the conversational frequencies (0.5, 1, 2 and 4 kHz) was measured for each ear, and its value for the best ear was used to define the severity of deafness: mild (20 dB < ACPTA ≤ 39 dB), moderate (40 dB < ACPTA ≤ 69 dB), severe (70 dB < ACPTA ≤ 89 dB), or profound (≥90 dB).

### Exome sequencing and Sanger sequencing

Whole-exome sequencing and bioinformatic analysis were performed as previously described [7]. Specific oligonucleotides were designed to PCR amplify and sequence the 21 exons of *EPS8* by the Sanger technique, using Primer3 (http://http/frodo.wi.mit.edu/primer3/) (Additional file 1: Table S1). EPS8-3 F forward primer: 5′-CGTGGTGATTATAGTGCATTGG-3′ and EPS8-3R reverse primer: 5′-ATCACTGCCTCATTCCAAAC-3′ were used to amplify and sequence *EPS8* exon 3.

### Immunofluorescence labeling

Dissection and preparation of mouse cochleas were performed as previously described [7]. Eyes obtained from deeply anaesthetized adult macaques perfused with paraformaldehyde, were dissected and processed as previously described [8]. Retinal cryosections were incubated in 10% bovine serum albumin in phosphate buffered saline (PBS) for 1 hour, incubated with the appropriate primary antibody overnight at 4°C, rinsed in PBS, incubated with the appropriate secondary antibody for 1 hour at room temperature, and rinsed again in PBS. The primary antibodies used were anti-EPS8 (BD Transduction Laboratories), anti-synaptophysin (MAB368, Millipore), anti-whirlin (G-8, Santa Cruz Biotechnology, Inc) mouse monoclonal antibodies, and anti-EPS8 (M-238, Santa Cruz Biotechnology, Inc) rabbit polyclonal antibody. Secondary antibody was Alexa Fluor 488 goat anti-rabbit IgG. TRITC-phalloidin (Sigma-Aldrich) and DAPI (1 μg/ml; Sigma-Aldrich) were used to label F-actin and cell nuclei, respectively.

## Results and discussion

Patients IV.1 and IV.2, who are twelve and eight years old, respectively, were born to first-cousin parents (Figure 1B). Both patients, initially mute, were implanted two years ago. Auditory brainstem responses to click stimuli of various intensities were recorded: no response to these stimuli was detected. More specifically, an elevation of hearing thresholds beyond 120 dB was found for both ears, for pure tones at all frequencies analyzed (250 Hz to 8000 Hz; Figure 1C) indicating profound deafness. Temporal bone CT scan revealed no cochleo-vestibular malformations. The patients did not start walking late (they both started walking at nine months) and did not have any balance problems. Clinical examination failed to detect additional symptoms indicating a syndromic form of deafness, and neither proteinuria nor hematuria was observed. Both parents and the three other siblings did not show any sign of hearing impairment, and audiometric tests carried out on the parents and one of the siblings revealed an absence of hearing threshold elevation for all frequencies tested.

We first sought mutations in the genes most frequently implicated in DFNB: *OTOF* and *MYO15A.* No mutations of these genes were found in the two patients. We therefore carried out whole-exome sequencing, with pooled genomic DNA from the two affected children, as previously described [7]. We excluded variants present in the dBSNP132, 1000 genomes, and HapMap databases, with a prevalence greater than 0.01%. As the parents were consanguineous, we hypothesized that the causal mutation would be present in the homozygous state in the patients. We first searched for sequence variants of the coding and non-coding exons and of splice sites in the known DFNB and DFNA (autosomal dominant non-syndromic deafness) genes. No such variants were found (none were detected in the heterozygous state either). We then focused on nonsense, missense, splice site and frame-shifting mutations in all genes, by seeking insertions/deletions and SNPs. By using the same criteria as above, the number of sequence variants found decreased from 6268 to 0 for insertions/deletions (total number of insertion/deletion variants found in this pooled DNA), and from 74007 to 2 for SNPs. One of these biallelic variants was a conservative missense mutation, c.1110A > C (p.E730D), in *BNC2* (gene ID: 54796, NM_01763), predicted to be nonpathogenic by PolyPhen-2, SIFT, and Mutation Taster software. The other was a nonsense mutation, c.88C > T (p.Gln30*), in exon 3 of *EPS8* (gene ID: 2059, NM_004447.5). An analysis of the segregation of this mutation, based on sequencing of the corresponding DNA fragments in the parents, the two affected children and three unaffected siblings, confirmed the biallelic mutation in the two deaf children and showed that the mutation was present in the heterozygous state in both parents and one sibling, but not in the other two siblings (Figure 1B, D). This mutation was not present in 120 Algerian control individuals or in the Exome Variant Server database. The mutation is expected to result either in an abortive protein truncated at amino acid position 29 or in no protein at all due to nonsense mediated mRNA decay [9]. It is therefore functionally equivalent to the knock-out of the orthologous gene in *Eps8*^−/−^ mice, which are also profoundly deaf [10,11]. We thus concluded that this mutation is responsible for the profound congenital deafness in the patients. The 39 remaining DFNB families were also investigated for the presence of mutations in *EPS8*, and we did not find any.

EPS8 is a 822 amino acids protein composed of a pleckstrin homology (PH) domain, a SRC Homology 3 (SH3) domain and a F-actin binding domain (Figure 1E). The C-terminal region has significant sequence homology with the sterile-motif (SAM) domains [12]. Overexpression of *EPS8* is associated with some human cancers [13]. The three EPS8-like variants EPS8L1, EPS8L2, and EPS8L3, encoded by different genes, together with EPS8, constitute the EPS8 family of actin regulatory proteins. Of note, mutant mice lacking EPS8L2 are subject to progressive hearing loss [14].

**Figure 2:**
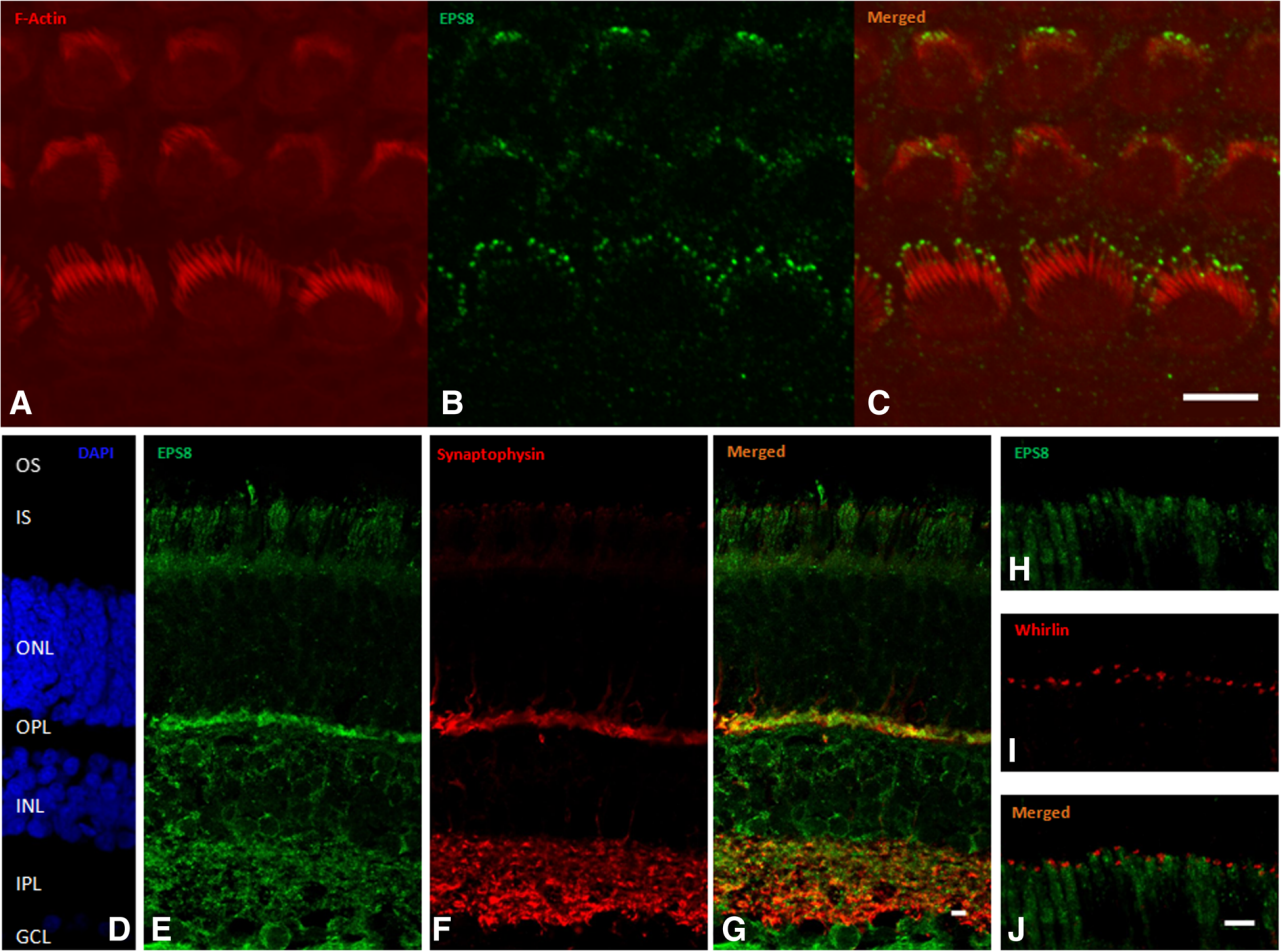
**Immunolocalization of EPS8 in the mouse cochlea and macaque retina. (A-C)** Immunolabeling for F-actin **(A)** and EPS8 **(B)** in the mouse cochlea. DAPI **(D)** was used to label cell nuclei. Immunolabeling of EPS8 **(E and H)**, synaptophysin **(F)** and whirlin **(I)** in the macaque retina. A polyclonal antibody was used to detect EPS8. **(G)** Co-immunolabeling of EPS8 and synaptophysin **(J)** Co-immunolabeling of EPS8 and whirlin. Abbreviations: OS, outer segment; ONL, outer nuclear layer; OPL, outer plexiform layer; INL, inner nuclear layer; IPL, inner plexiform layer; GNL, ganglion nuclear layer. Scale bars: 5 μm.

The mechano-electrical transduction of sound stimuli takes place in the two types of auditory sensory cells, the inner and outer hair cells of the cochlea [15,16]. The transduction process involves mechanically gated ion channels located at the tips of specialized, stiff microvilli known as stereocilia. These stereocilia are organized into three rows of increasing height, and together they form the hair bundle projecting from the apical surface of the hair cell. The stereocilia have a cytoskeletal core composed of tightly packed actin filaments. Hair bundle development involves tight control of the differential elongation and thickening of stereocilia [17]. EPS8 is present at the tips of the stereocilia of the inner and outer hair cells (see Figure 2A-C), where it forms a tripartite molecular complex with MYO15A and the PDZ-domain containing protein whirlin [10], both of which are involved in the control of stereocilia length [10]. EPS8, a F-actin capping and also a cross-linking or bundling protein, is involved in F-actin dynamics [18], and knockout mice lacking EPS8 [10,11], like MYO15A- [19] or whirlin [20]-deficient mice, have abnormally short hair cell stereocilia. In addition, inner hair cells of *Eps8*^−/−^ mice show a defective maturation [11].

Because mutations of the whirlin gene, *WHRN*, can cause Usher syndrome of type 2 (USH2D), which associates retinitis pigmentosa to the hearing impairment, we looked for clinical features of retinal defect in the two patients. They did not present defective vision in low-light conditions and ophthalmologic examinations, including fundus autofluorescence, gave normal results (data not shown). As the patients are still young and the abnormal retinal phenotype of USH2D usually appears later in life than in other genetic forms of USH2 [21], we cannot definitively rule out the possibility of a syndromic deafness phenotype. We thus investigated the possible interaction of EPS8 and whirlin in the retina, by assessing the colocalization of these two proteins. Mouse models of Usher syndrome do not faithfully reproduce the visual impairment observed in humans. We therefore assessed colocalization in the adult macaque retina, by immunolabeling experiments based on a technique previously described [8]. Whirlin was detected at the junction between the inner and outer segments of the rod and cone photoreceptors, around the base of the connecting cilium, in the periciliary membrane complex region, as previously reported [8,22], whereas EPS8 was present in small amounts in the inner segment of rod photoreceptors, but was not colocalized with whirlin. By contrast, strong EPS8 immunolabeling was associated with the inner plexiform layer (consisting of the processes of bipolar cells, ganglion cells, and amacrine cells) and the outer plexiform layer of the retina (consisting of the processes of rods and cones, horizontal cells, and bipolar cells), in which it was colocalized with the presynaptic protein synaptophysin. The same EPS8 labeling was observed with two different antibodies (a monoclonal and a polyclonal antibody) directed against the protein (Figure 2 and data not shown). The lack of whirlin and EPS8 colocalization in macaque photoreceptor cells indicates that these two proteins, which interact directly in the hair bundles of cochlear hair cells, are unlikely to interact in the retina. These results are not consistent with mutations in *EPS8* causing an Usher-like phenotype in patients, and instead identify this gene as responsible for a new autosomal recessive form of isolated deafness.

## Conclusion

To conclude, we report for the first time the identification of one biallelic nonsense mutation in *EPS8*, in two children affected by profound congenital deafness. This new DFNB form is likely to arise from abnormal hair bundles resulting in compromised detection of physiological sound pressures.

## Abbreviations

EPS8: Epidermal growth factor receptor pathway substrate 8; DFNB: Nonsyndromic deafness, autosomal recessive; DFNA: Nonsyndromic deafness, autosomal dominant.

## Competing interest

The authors declare that they have no competing interests.

## Supplementary Material

Additional file 1: Table S1. Primers for PCR amplification of *EPS8* exons Click here for file
