# Supplementary material for: EPS8, encoding an actin-binding protein of cochlear hair cell stereocilia, is a new causal gene for autosomal recessive profound deafness
Source: Orphanet J Rare Dis. 2014 Apr 17;9:55. doi: 10.1186/1750-1172-9-55 (PMC4022326; doi:10.1186/1750-1172-9-55)
Supplement: Additional file 1: Table S1. — Primers for PCR amplification of EPS8 exons [file 1750-1172-9-55-S1.docx]

**Additional file 1: Table S1**. Primers for PCR amplification of *EPS8* exons

|  | Forward (5'-3') | Reverse (5'-3') |
| --- | --- | --- |
| Exon 1 | GCCTTGGTCCGACTTTGATC | AGCACACAAAGGCGGATTTT |
| Exon 2 | TGAGCTCTTTGAGGGTGGAG | CAGAAACATCTTCCAAGTGTGAG |
| Exon 3 | CGTGGTGATTATAGTGCATTGG | ATCACTGCCTCATTCCAAAC |
| Exon 4 | CACAATGGCAACTTGTCAGC | TCATTCTTTATGAAAATAGACCACAG |
| Exons 5-6 | TTTGGAAAATGTATACTAAGAGGTTG | AAAGCTCCCAGACAATCTGC |
| Exon 7 | GATTCAGACAAGGAACAATCCC | GAACAAATGTGTTGAGACTACAGG |
| Exon 8 | GGAAATCCTTATTGTCCCTTGAC | GCAAGAACTATGTCCCAACCC |
| Exon 9 | TATCCAGTGGAGGTGTTCCC | ATTCAAAGGGCATAGCCATC |
| Exon 10 | ATGTGGGCTGCTTCCTTTTC | CCTTAGATGATACACCCGCC |
| Exon 11 | AAGGATAGAGCAATCCATTTTG | GCATGGATAGAGTGAGAATAGAAGTC |
| Exon 12 | TCTGGTGGTAAAGACCGTCC | ACCCACGCAAGGTAATGAAG |
| Exon 13 | GTAGCACATTGCACAGCATC | CCTATGACGCTTAGTCCTTCG |
| Exon 14 | TGTCTAGCCTTACTGAAGATAACTGTG | CAGTTGAATAAAATGAGAACTTGC |
| Exon 15 | TGACTGACCTGAGTGCTGATTC | AACAGATGGAAAGTTTAAAGAAGC |
| Exon 16 | TGGCTTGTCTCAGTGTTTTCC | AAACCCACCCTGCCACTC |
| Exon 17 | GGGAACTTCTTTCGTAGAATGG | CTGTGTCTCTCACCATTGCC |
| Exon 18 | AGCCAACAATGTCCTATCAAAG | TTAAGTACCATGCAAGTATAAAGGAC |
| Exon 19 | AGGTCCTACTGGGGTCAAGG | CAAAGTATGCAGTCTGTGCCC |
| Exon 20 | GAAAAGAGAAGTTACTAGCAGGAACAG | TGTTAAGAAAGGGAAATTGGC |
| Exon 21 | TCATGATATGACCTGCAGCC | CTCACTCAGGTTTGCATGGG |
